# Taking a deep breath: a qualitative study exploring acceptability and perceived unintended consequences of charging clean air zones and air quality improvement initiatives amongst low-income, multi-ethnic communities in Bradford, UK

**DOI:** 10.1186/s12889-021-11337-z

**Published:** 2021-07-03

**Authors:** Rukhsana Rashid, Felisha Chong, Shahid Islam, Maria Bryant, Rosemary R. C. McEachan

**Affiliations:** 1 Bradford Institute for Health Research, Bradford Teaching Hospitals NHS Trust, Bradford, BD9 6RJ UK; 2 Department of Health Sciences and the Hull York Medical School, University of York, Heslington, York, YO10 5DD UK; 3 Faculties of Life Sciences & Health Studies, University of Bradford, Richmond Road, Bradford, BD7 1DP UK

**Keywords:** Air pollutants, Clean air zone, Air quality, Seldom heard, Health inequalities, Bradford, Environmental justice, Traffic, Transport, Cycle lanes

## Abstract

**Background:**

Poor air quality is the one of the biggest causes of early death and illness across the lifespan. In the UK, 28 local authorities with illegal pollution levels have been mandated by the Government to develop plans to rapidly reduce pollution to legal limits. These plans include consideration of implementing one of four of charging 'Clean Air Zone’ (CAZ) classes in areas of high pollution which would charge older polluting vehicles a daily charge to enter. While this offers a potential to improve air quality, the extent to which CAZ might impact (for example, economically) on socio-economically deprived groups and local businesses is unclear.

**Aims:**

To explore the acceptability and perceived unintended consequences of a CAZ and other initiatives to improve air quality with seldom-heard communities living in deprived, multi-ethnic areas within the city of Bradford, UK.

**Methods:**

Ten semi-structured focus groups were conducted with people who live in areas of high pollution and deprivation. A total of 87 people participated from a diverse range of ethnic backgrounds with the majority of Pakistani origin. Recorded data were transcribed, coded and analysed using thematic analysis.

**Findings:**

As poor air quality was not always visible it was seen as a hidden issue by many, and not prioritised over other more visible environmental issues (e.g. fly-tipping, littering). There was resistance to proposals which included charging private vehicles. Many felt that low-income families did not have the resources to purchase compliant vehicles or pay daily charges, placing a disproportionate burden on them. It was also felt that low-income taxi drivers would be disproportionately affected financially by proposals. Public transport infrastructure was felt to be inadequate. Other traffic management or emission reduction activities were also explored. Views towards these initiatives were more positive if they did not directly affect individuals financially.

**Conclusion:**

Air quality initiatives such as CAZs were felt to be likely to financially disadvantage communities already living in socio-economic and environmental poverty. Policy makers need to carefully consider appropriate mitigation strategies to ensure that health and economic inequalities are not increased by implementation of CAZ. Given air quality is low priority for some groups, careful engagement and communication will be required to increase acceptance interventions such as CAZs.

**Supplementary Information:**

The online version contains supplementary material available at 10.1186/s12889-021-11337-z.

## Background

Air pollution is one of the biggest contributors to mortality and morbidity globally [1]. Research has linked poor air quality with a range of health outcomes, including poor birth outcomes such as low birth weight or preterm birth [2]; cardiorespiratory disease [3]; lung [4] and non-lung cancer [5] cognitive development and neurological disorders [6]. The costs of dealing with illness related to air quality are substantial. Between 2015 and 2035 the forecasted costs to the UK NHS for air quality related illnesses is estimated to be £5.4 billion [7], with the wider economic cost to society estimated to be £20 billion/year [8].

All the available evidence points towards a case of environmental injustice as the burden of exposure to poor air quality is disproportionately borne by those of lower socio-economic status (SES) [9, 10]. Further, communities that experience poor health across a range of measures are likely to find health inequalities compounded due to poor air quality [11, 12].

The UK is currently breaching legal limits of key pollutants such as Nitrogen Dioxide ([NO_2_]_,_ annual mean of 40μg/m3, [13] and regularly exceeds World Health Organisation (WHO) guidance for Particulate Matter (PM) [14]. Sixty per cent of the UK population live in areas which exceed poor air quality thresholds, [15] and one third of children are exposed to unsafe pollution levels [16]. As a result, the UK Government has issued ministerial directions to 28 local authority areas [17] to reduce pollution in the quickest time possible.

‘Clean Air Zones’ (CAZ), impose a charge for older more polluting vehicles entering specific areas (petrol vehicles below the Euro 4 standard implemented in 2005, and diesel vehicles below the Euro 6 standard implemented in 2014). This approach has been identified by the UK Government [18, 19] as potentially effective in reducing air pollution [20, 21] and thus improving health [22, 23]. There are four classes of Clean Air Zone recommended for consideration: a Class A CAZ restricts charging to non-compliant taxis and buses; Class B includes A, in addition to heavy goods vehicles (HGVs); Class C extends A and B to also include light goods vehicles (LGVs) and Class D includes all of the above in addition to private vehicles which are not compliant with Euro 6 (diesel) and Euro 4 (petrol). It is suggested that these zones be supplemented by a range of additional activities which aim to further reduce emissions; for example, promotion of electric vehicles, park and ride schemes, promotion of active travel and retrofitting older polluting buses to compliant standards.

However, interventions to improve air quality can have unintended consequences. For example, researchers have found that despite 16 years of efforts to improve air quality in the UK, improvements have been greatest in more affluent areas, and yet in more deprived areas air quality has continued to deteriorate, widening health inequalities [24, 25]. Policy interventions to improve air quality which involve penalising or restricting drivers who drive older, more polluting vehicles may disproportionately affect poorer communities who may be more likely to own older used vehicles [26] and therefore less likely to own compliant vehicles. A prospective health impact assessment of the London Low Emission Zone [27] (implemented in 2008) identified potential adverse outcomes on smaller businesses and voluntary or community sector organisations, who would struggle to cover costs of upgrading vehicles to compliant standards, which, in turn, could have a potential knock-on impact on employment and provision of voluntary or community sector services amongst vulnerable communities.

It is important to fully explore the potential adverse consequences from implementing policy interventions such as charging clean air zones prior to implementation to ensure that they do not inadvertently disadvantage certain groups, or widen existing inequalities. Identification of potential unequal impacts affecting disadvantaged groups during policy development means that mitigating strategies can be put in place to address these adverse impacts [28]. Local communities, who will be affected by policy changes, are equally or better placed than policy makers to identify potential positive and negative outcomes of policy interventions.

The City of Bradford, in the UK, is one of the 28 local authorities identified as having illegal level of pollution, and has subsequently been directed by the Government to develop a ‘Clean Air Plan’ including consideration of implementing a charging clean air zone to reduce pollution levels as quickly as possible. The aim of the current study was to explore the acceptability and potential unintended consequences of implementing a charging clean air zone, along with other air quality initiatives within this area by engaging 'seldom heard’ multi-ethnic communities living in deprived areas with a view to informing development of Bradford’s ‘Clean Air Plan’. We also aimed to explore how communities perceive and understand the relationship between air quality and health.

Our research questions were: 1) How do multi-ethnic communities living in deprived areas perceive and understand the relationship between air quality and health? 2) What is the acceptability and what are the potential unintended consequences of implementing a charging clean air zone for these communities? 3) What do communities think about a range of other supplementary air quality initiatives?

## Methods

### Design

We conducted a qualitative study, including focus group discussions to explore participants’ views around air quality, and reactions to the potential introduction of different CAZ ‘classes’ (Table 1). Groups were also asked to discuss a range of additional measures being considered by Bradford Council which could be implemented in addition to a potential CAZ. These were loosely categorised into ‘Emission Reduction’ activities (e.g. activities which directly cut levels of emission by replacing buses and taxis with electric vehicles) and ‘Travel and Transport Management activities’ (e.g. activities designed to reduce emissions by altering flow of traffic on the road through improvements in highway). Finally we explored reactions to individual behaviours which could improve air quality such as upgrading to electric vehicles and increasing active travel or public transport (a full list of CAZ classes and other initiatives which were discussed can be found in Table 1).

**Table 1:** Description of Clean Air Zone ‘Classes’ and other key initiatives under consideration by Bradford Council

Initiative classification	Description
Clean Air Zone Class A	A charging zone to include non- compliant buses, coaches and taxis
Clean Air Zone Class B	A charging zone to include non- compliant buses, coaches and taxis, and heavy good vehicles
Clean Air Zone Class C	A charging zone to include non- compliant buses, coaches and taxis, heavy goods vehicles and light goods vehicles.
Clean Air Zone Class D	A charging zone to include non- compliant buses, coaches and taxis, heavy good vehicles, light goods vehicles, private cars and motorbikes
**Emission reduction initiatives**	
Green buses scheme	This involves bus companies replacing their current fleet with greener vehicles.
Installation of electric charging infrastructure in new developments	The aim of this proposal is to encourage installation electric car charging points in new housing / planning developments to encourage people to consider electric vehicles in the future.
Council fleet improvements	The city council own a large fleet of vehicles from rubbish collection lorries to smaller vans and other vehicles. This initiative would involve the council replacing their fleet with greener vehicles
**Travel and transport management**	
Park and ride	A park and ride scheme was proposed that could see drivers coming into the city park their cars at a designation outside the city centre and then complete their journey on public transport.
Road widening/ highways improvements	This proposal would consider widening some busy roads around the city to improve traffic flow and reduce traffic build up.
Intelligent transport systems	The proposal for Split Cycle Offset Optimisation Technique (SCOOT) [29] is a method of adaptive signal control where vehicles are detected as they approach a signalised junction well in advance of the stop line and can help drivers adapt their speed accordingly when approaching junctions to minimise a need for stopping which then causes idling. The detection, from multiple junctions, is fed into a central system, which models the flow of traffic in the area.
Increasing city centre parking charges	Parking charges in many public carparks in Bradford (and particularly in the city centre) are amongst the lowest compared to the national average. In some respects therefore it is more cost effective to drive and park rather than use public transport. This initiative was considered to help reduce the number of people driving into the city centre.
Freight consolidation centres	This involves building consolidation centres where large HGVs for stock distribution in the city can arrive and unload and then have goods taken out and distributed in smaller greener vehicles. This would reduce the number of HGVs on the highways.
HGVs- traffic diversions and delivery times	Participants considered whether HGVs should be restricted to using the highways at certain (non busy) hours of the day to reduce pollution levels and traffic at busy times.
Travel planning	This proposal was to consider people car sharing where neighbours or colleagues were travelling to the same/ similar destinations.
Cycling and cycling infrastructure	Although there is already cycling infrastructure in Bradford, this initiative proposed improvement to these and promotion of wider use by local residents.
**Additional ideas explored**	
Electric cars	Participants explored the barriers and facilitators of a shift towards electric personal vehicles.
Public transport and active travel	Participants considered the barriers and facilitators of more public transport use and active travel methods.

Notes: for purposes of interviews estimated charges for the CAZ were as follows: £6.00 for private vehicles and motorbikes, £12.50 per day for private cars and taxis, £9.00 for LGVs and vans, and £50 per day for buses, coaches and HGVs

A semi structured topic guide was devised to help lead the discussion covering key topics including: general knowledge about air pollution and health; the acceptability, feasibility and barriers to implementation of the different CAZ classes and other initiatives under consideration; potential unintended or adverse impacts of the CAZ and other initiatives and community ideas about other ways to reduce pollution (see Supplementary File). Visual aids were used to help explain each of the CAZ classes. The study was approved by University of Bradford Ethics committee (E702, amendment 2, 16th June 2019) and conducted in accordance with the Declaration of Helsinki.

### Setting

Our study was located in Bradford, an urban, multicultural city in the North of England, UK. Bradford is the 5th largest metropolitan district in England with a population of > 530,000 [30]. It has a multi-ethnic population with the last census data from 2011 showing that 63.9% of the population identified as White British, 26.8% as Asian/Asian British (predominately of Pakistani heritage); 2.5% as from mixed multiple ethnic groups, 1.8% as Black/African/Caribbean/Black British, 0.5% identifying as White Irish, 0.1% identifying as Gypsy or Irish Traveller; 3% as an ‘other White’ group and 1.5% as an ‘other ethnic group [31]. It is a socio-economically deprived city, with 40% of Bradford residents living in areas that rank in the most deprived quintile (20%) of local areas in England [32]. It has high levels of ill health; for example, with higher than average (in relation to England) mortality from cardiovascular disease under 75 years (102.2 per 100,000), low birth weight babies (3.6%) [33], and 22% incidence of wheezing disorders amongst children [34].

Figures obtained from Bradford Metropolitan District Council for 2020 evidenced the availability of 207,500 registered cars within the district. This is approximately 69% of households in Bradford. For the same year there were 3741 registered taxis and 5000 registered taxi drivers. Of these, 428 were hackney carriages with the rest being private hire (Personal Communication).

**Fig. 1:**
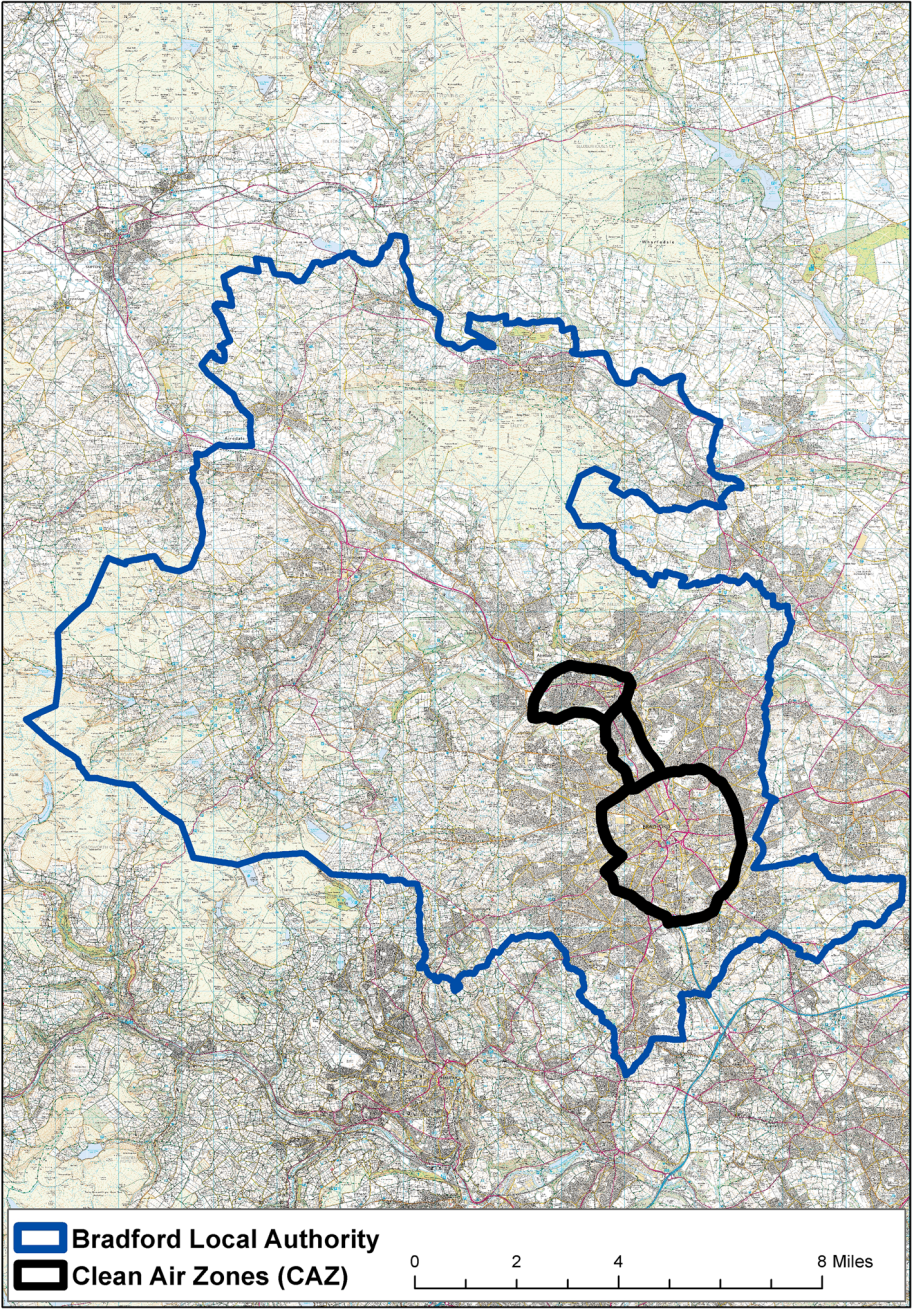
Indicative CAZ boundary in relation to Bradford District

The study was set across seven electoral wards, selected as they have highest levels of pollution in Bradford district. Six wards fell within the proposed CAZ boundary area (Fig. 1), and one ward fell outside of the boundary area. The wards are comprised of various ethnic identities. According to the 2011 census, three of the wards had a majority of residents who identified as Pakistani, with White ethnic groups making up a small proportion of the overall number. One of the wards had equal proportions of both Pakistani and White ethnic groups, and the remaining three wards were comprised of residents who mostly identified as belonging to White ethnic groups; in these wards Pakistani residents constituted a minority (see supplementary file).

### Sample

Adults over the age of 18 who lived or worked in the seven selected electoral wards in Bradford were invited to participate. Ten focus groups with *n* = 87 participants were conducted. Discussions were conducted in a range of languages including English (six groups), Urdu (four groups); Urdu/Bengali (one group), and Slovakian (one group). We planned to recruit focus groups until we reached saturation, ensuring these was at least one focus group in each ward.

### Recruitment and procedure

Participants were recruited with the help of community organisations and community connectors. Community connectors were ‘trusted’ community members who worked at local community organisations, community centres or other voluntary sector organisations in the areas and had established trusted relationships within communities. As such, their role was central to identifying and inviting participants to the research, and to creating trust with the research team. They also helped organise the focus groups sessions and sometimes took notes. As community members themselves we felt their views were equally important, therefore they were invited to take part in discussions if they wished and, in this case, were treated in the same way as other participants.

Community connectors invited participants to take part in the research and provided them with an information sheet. Due to the nature of the initial approach via community connectors, refusals or lack of interest were not formally recorded. A date, venue and time was agreed between the participants and the community connectors on behalf of the researchers. Participants were given the opportunity to ask any questions and asked to provide informed consent (facilitated by the researcher) before the focus group discussions began. Focus groups were semi-structured and, lasted approximately 90 min. The sessions took place at accessible local venues in each ward including community centres, libraries and schools at different times of the day to increase participation and representation. Community connectors were present during the focus group discussion and also participated. To overcome cultural sensitivities amongst some ethnic groups, we included some single gender focus groups (2 male and 2 female), and 4 mixed gender groups.

The sessions were audio recorded and later transcribed verbatim. A member of the research team also took field notes at each of the sessions. The majority of the focus groups were conducted in English; however two were conducted in both Urdu and English by a multi-lingual researcher and one was conducted in Slovakian through the use of an interpreter. Participants were offered refreshments and a £10 reimbursement to cover expenses.

The focus groups were facilitated by RR who has over 15 years’ experience in conducting and analysing qualitative research with ethnic minority groups. The interviewer has an MPhil and was employed as a research fellow at the time working on projects related to air quality and health. The interviewer was supported in some of the focus groups by a second researcher who took field notes during the sessions. The researchers had no existing working relationships with the community connectors (also participants), who in turn had a ‘service provider’ relationship with other participants.

### Analyses

Focus group data were analysed using thematic analysis [35]. Themes were generated using an inductive approach and a coding framework was developed around this. These were subject to continuous updates as the data was read. The themes and codes were developed using the one sheet of paper (OSOP) method [36]. This involves reading through transcripts and noting codes on a single sheet of paper, linked to participant’s accounts, before combining these codes into broader themes. Data were first coded by (RR) and double coded by (FC) using NVIVO software. Themes were agreed between the two coders and any differences were resolved after discussion.

For the specific items discussed in the proposals (emission reduction and transport and travel management initiatives), content analysis [37] was used to describe the acceptability and feasibility of each.

## Results

Of the 87 recruited participants 35 were male and 52 were female. The mean age of participants was 38 years (range 20–70 years). The majority of participants were of Pakistani origin (*N* = 71), with representation from White British (*N* = 4), Indian (*N* = 4), White European (*N* = 3), Bangladeshi (*N* = 2) or other ethnic group (*N* = 3), a full breakdown of ethnic demographics can be found in the supplementary file. Through discussion, a large proportion of respondents reported that either their own occupation or a family member’s occupation was as a private hire vehicle or hackney carriage taxi driver.

### General knowledge and beliefs about air quality

Participants had varied degrees of knowledge and interest about air quality in the areas where they lived and worked. Many reported being aware through increased media coverage of air quality and plans to implement clean air or low-emission zones in other parts of the country. Participants reported some awareness about the impact of poor air quality on health, and in particular respiratory conditions.

*“When the weather’s bad and we’ve got low cloud coverage, like everywhere else it keeps that layer hanging around in the atmosphere and the particles are so small anyway anybody with breathing problems, asthma, COPD, anything like that, and then the children become allergic to different things, they have more incidents of developing asthma, and other breathing problems”* *(FG1- White British, Male, Retired).*

However, a few participants were sceptical about the extent of poor air quality in their area as they felt that it was not something that could be quantified and measured. *“I mean it’s something you can’t measure can you really, but judging by what we get told and the sort of emissions that are flying about in cars and stuff, I’d say it’s very poor quality but then how do you know, you can’t see it” (FG7- British Asian (Pakistani), Male, Industrial worker).*

Nevertheless there were some people who felt the difference in air quality was noticeable in different parts of the district with rural more affluent areas having cleaner air.

*“I normally go on Sundays, we go out on the outskirts of Bradford, go to Harrogate and that area, that you can see, that you can feel that the air is different … Inner city areas like we’ve got here, we’ve got for example Girlington, Leeds Road areas, that’s breathing in disease but then you’ve got places like Bingley* [more affluent areas in Bradford district] *who have less traffic, less pollution, more greener spaces, they don’t seem to have that same level of respiratory disease, so it’s all linked” (FG7- British Asian (Pakistani), Male, Sports coach).*

As a post-industrial northern town, some participants as residents in urban parts of Bradford felt that, although traffic was a major contributor to air pollution, it was not the only factor. Large factories and takeaways, particularly in highly populated deprived neighbourhoods in Bradford were seen as a significant contributor. *“In addition to the traffic we are located in an area which is partly residential and partly industrial, so on the other side of Leeds Road it’s all industrial area, and the smoke from the chimneys, from industrial businesses is huge” (FG1- British Asian (Other), Male, Bus driver).*

*“We’ve been concentrating on transportation pollution, and the occasional businesses, but with all the takeaways how much gubbins is coming out of their vents from all these various cooking establishments, on here, it’s ridiculous, because sometimes you’d be sat in the house with all your windows and doors shut and you think oh crumbs what’s cooking now because it stinks” (FG1- White British, Male, Retired).*

There was a mixed level of concern about air quality in local communities, with some caring about the issue, whilst others being either unaware or unconcerned.

*“I think people do realise it but they don’t really, care about with the pollution and what they’re doing. Because if you look at some of, like the cars and stuff round Bradford, a lot of them, you know, they drive around with all this black stuff coming out and they use all these petrols and oils which are bad. All this red petrol and stuff that they use in the cars and that’s really bad for the air and they know that it is and it’s like they have this attitude of like “we don’t care”, you know, “we don’t care about the community or the environment or the pollution or whatever it’s causing”. For them it’s showing off their car” (FG2- British Asian (Pakistani), Female, Housewife).*

### Other environmental concerns

For the majority of participants, living in neighbourhoods where there are very high levels of deprivation, other more visible environmental issues were of greater concern to them. These issues, it was felt, affected their day to day lives more significantly than poor air quality and therefore needed more urgent attention:

*“You see a lot of rubbish, cigarette ashes and all sorts of things. There’s not enough rubbish bins for people to dispose their rubbish. And I think it doesn’t help the fact though when people have got, you know, fortnight bin collections as well and sometimes you see overflowing bins of rubbish. So all these things obviously contribute to, you know, the air quality and what have you” (FG6- British Asian (Pakistani), Female, Community support worker).*

*“The drains are cleaned out regularly and you know when you’re walking past and the smell it’s really strong when it gets really bad. And that’s the only time when you’ve got to ring them [the council] and they come out and do something about it. But that smell, it’s like the dead rat and it happens regularly and this is what we have to live with. For me, living with this is harder than pollution … and the rubbish and fly-tipping. These are the real issues (FG1- British Asian (Indian), Female, Retired).*

Participants felt that discussion around greenery and green spaces was missing from the proposals to improve air quality. *“The other thing I feel Council could do is maybe plant more greenery” (FG6- British Asian (Pakistani), Female, Unemployed).* They felt that the lack of green spaces in their neighbourhoods contributed to the higher levels of air pollution. Thus, a more holistic approach to cleaner air was felt more appropriate than solely focussing on vehicle pollution, which some interpreted as penalising an already disadvantaged community instead of investing in it.

Some participants felt that the priorities of the community are ignored and overlooked in favour of policies that they perceived would have little benefit to them, such as a CAZ. This is not surprising given the visible disadvantage and deprivation amongst these communities to whom air quality appears as a ‘middle-class’ issue.

*“People in these areas are very poor. They live on small wages or on JSA [*jobseekers allowance*]. I have to rely on this charity* [session was held at a women’s community mental help support centre]*. Sometimes I have to decide if I should eat or if I should pay the bills. We’re the wrong people to ask about these things. Owning a car is a luxury. In Keighley we battle to survive” (FG6- British Asian (Pakistani), Female, Unemployed).*

### CAZ proposals

Participants had a range of detailed and in-depth thoughts and opinions on the acceptability, deliverability and feasibility of the CAZ proposals as well as wider initiatives targeted at improving air quality.

There was no consensus across focus groups about which CAZ option would be most acceptable. Opinions appeared to be based on individual characteristics including age, gender and how people travelled. For example, the majority of over 65 s preferred class D (including private vehicles) *“as it definitely seems like the best one [all agree]” (FG1- White British, Male, Retired)* because they were more likely to use public transport and felt that they were therefore less likely to be affected by charges.

Some participants also felt implementing a CAZ D could have additional benefits in relation to reducing volume of traffic on the road. Of particular concern was dangerous driving caused by some road users and more generally a high number of vehicles on the highways. This was particularly a concern for older participants who felt more vulnerable as pedestrians. They felt that a class D CAZ would discourage people from driving for short journeys.

*“You’ve got parents double parked, triple parked, with their engines running waiting for their little particular darlings to come trotting out of school which is 2 min down the road. There is no need for it. You get so many idiots in Bradford that ignore all road signs, speeding there is a road safety issue of the way people drive, they will go through red lights (FG1- White British, Female, Retired).*

On the other hand, participants that owned cars which did not meet compliant emission levels for the CAZ were more likely to prefer one of the other classifications so to avoid being personally penalised. However, there were very few participants in favour of class A (targeting only non-compliant taxis and buses) as people saw this as having only a modest impact on reducing air pollution.

There were suggestions that a gradual introduction of the CAZ classifications (starting with the implementation of A and moving up to D) would be more favourable with the community as it would give people the opportunity to get accustomed to having a CAZ and give them time to make necessary arrangements. It was argued that this would increase acceptability and reduce the burden that some households may face.

*“I’m just giving examples, so for example you’re starting with Class A, that you put in for maybe a year, 18 months, so everyone gets ready for that, for example like this gentleman’s a taxi driver here, he does it for his living, you’re just going to hit him where it hurts so they might not be able to deliver, but the point is if you gradually think that they can make lifestyle changes, or changes towards that, hold on a minute, like we have to think more eco-friendly, so then they might start getting hybrid cars and so on” (FG7- British Asian (Pakistani), Male, Youth worker).*

There was an awareness amongst members in this community about the social injustices that they face on many levels which made them question perceived ‘hidden agendas’ of any new initiatives implemented in their areas.

*“There’s always, the Council always gives planning permission for takeaways and for businesses in predominantly Asian areas. You will never be able to get planning permission for Baildon or Ilkley* [rural/ affluent areas in the Bradford district], *for those kind of, you know, businesses, you’ll never get it. They won’t allow it whereas Asian areas which are already very densely populated and has a lot of air pollution, they think it’s okay, they’ve already got some, so it won’t make a difference to them. I think they need to look at being, not being biased because they are very biased depending which area you are in” (FG6- British Asian (Pakistani), Female, Housewife [husband is a taxi driver]).*

Some people also felt that the disadvantages of introducing a clean air zone in their community outweighed any potential benefits. Financial factors were a key driver of opinions, and this is discussed in more detail below.

Many participants felt there should be more public consultations about any new CAZ initiatives to give people the opportunity to understand and better prepare for the eventuality that for some people can have a profound impact on their day to day life. It was argued that better public engagement would also lead to improve acceptability.

*“Just need to keep us in the knowhow, in the loop, and at the moment government being the way it is, they just seem to spring things on, “this is what you need to do”, not giving us an option, “would you like to do it?” you know. They need to tell us how we need to do it, when we need to do it, [otherwise] people panic. So they need to let us know well in advance that “yeah, we’re proposing this but you’ve got so many x, y and z months, this is where we suggest you go, this is how we suggest you do it”, you know, not just say like this is it” (FG5- British Asian (Pakistani), Female, Housewife).*

*“Because it’s actually a lot harder to change a decision, once something’s done it’s a lot harder but if you involve people then chances are sometimes they can offer you better solutions because they have knowledge of the local area” (FG6-Brithsh Asian, Female, Community support worker).*

Nevertheless, for some the plans to implement a CAZ which would primarily reduce emissions from vehicles was thus regarded with suspicion as participants felt that the motive of policy makers for a clean air zone was to financially benefit from penalising individuals rather than improve air quality. They felt that as plans do not address other perceived key sources of pollution or environmental injustices experienced within these communities. One participant said: *“if I speak my heart I would say this is the government is just trying to rip people off more” (FG7- British Asian (Pakistani), Male, Taxi driver).* There was also some discussion about how funds collected from the CAZ should be used. *“I think it’s a question that needs to be asked, the money that they’re going to make out of this are they going to use that to make the air better, how is that money going to be used will they invest it into making the environment in these areas better?” (FG7- British Asian (Pakistani), Male, Taxi driver).*

#### Financial implications

One of the biggest concerns that was consistently discussed amongst all participants was in relation to the financial implications. *“It’s going to affect everyone’s pocket really, it’s the public that’s going to get affected more than these companies, because they’ll just put their prices up and it’s people like us that will pay for it” (FG1- White British, Female, Retired).*

Many participants were concerned about the financial implications to businesses such as bus and HGV companies and how any charges or cost to upgrade their fleets could then be passed to consumers. *“You’ve got to think well then you’ll have the HGV drivers saying it’s affecting their business which means they pass the cost onto the businesses that use them, so they pass it on, so eventually whether you’re a driver or not, as a consumer you’re paying the bill for, so yeah” (FG1- British Asian (Other), Male, Bus driver).*

Participants overwhelmingly felt that more deprived communities would be disproportionately affected by a CAZ as they are already less likely to afford compliant vehicles:

*“Most people have got cars that are older than 2008, So you’re looking at people who are struggling, we’ve got a really high user food banks at the moment, we’ve got schemes on for children because they’re not getting meals at home because parents can’t afford to put three meals on the table for them. So how are these families going to pay these charges?” (FG3- British Asian (Pakistani), Female, Teacher).*

It was argued that the impact of these propositions without support for poorer communities would snowball into pushing people further into poverty and, in turn, affecting their health and wellbeing. A local charity worker gave the following account:

*“Because we have people coming here, even now with austerity we have people coming here, we have to provide food for them because of their, you know, difficulties, financial difficulties they have. So if you keep imposing penalties after penalties and further charges, then how are people going to survive? How are they going to manage? Do they not think that, you know, it’s going to affect their mental health, it’s going to affect their physical health and that is going to then impact on the NHS and other services” (FG6- British Asian (Pakistani), Female, Housewife).*

#### Disproportionate burden on taxi drivers

A large proportion of focus group participants were either private hire drivers or had a close family member that was a taxi/ private hire driver. This was not deemed to be unusual, as it is a common profession amongst low-income and socially deprived communities of predominately South Asian background [38]. For many, private hire vehicles were also used as families’ main household cars. The inclusion of taxis and private hire vehicles in all proposed classes of CAZ was met with strong opinion and concerns and it was felt that they would be negatively and disproportionately affected by the implementation of a charging CAZ. *“A lot of people who are in taxis are in taxis because they have no choice, they don’t have anywhere else to go, there’s nowhere else they can get jobs” (FG6- (British Asian (Pakistani), Female, Housewife).*

*“But the thing is you’re asking people who are struggling anyway, this is the people that’s been affected, we’re not asking about the middle-class, we’re talking about the people that are really deprived, right, where it’s affecting most, when they’re going “oh hang on” and a lot of people are taxi drivers as well, most of Bradford is taxi drivers, so it will affect a lot of people” (FG3- (British Asian (Pakistani), Female, Office worker [father is a taxi driver]).*

Many participants felt it was unfair to class private hire vehicles in the same category as hackney carriages or public transport. For example, private vehicles hire operators are predominantly self-employed and have a lesser earning potential as they are only licenced for pre-bookings. *“There’s a lot of taxis that are private hire, like the Uber drivers that use their own vehicle, but don’t have the same rights [as taxis] so why should they be treated in the same way?” (FG1- British Asian (Pakistani), Male, Council worker).*

There was also concern for taxi users that as self-employed; private hire drivers will inevitably “*increase their ticket prices” (FG1- Female).* This again would have the greatest burden upon some of the poorest households who cannot afford a car of their own and rely on public transport and private hire.

Many participants who were taxi drivers themselves suggested that they would be open to upgrading their vehicles or changing to electric cars if there was financial support to help them achieve this. Or they would wait until electric cars are cheaper as many lacked the initial capital to fund an upgrade unsupported:

*“It’s a good idea but I’ve just paid £8,000 last year for a diesel (private hire) car you see, that’s a bit of a waste of money for me now, because I am going to, I mean I don’t mind getting an electric car if the government’s going to somehow help me to buy that car, otherwise, I can’t until I’ve covered the cost of my first car. If there was help with the green cars then you can implement the change” (FG7- British Asian (Pakistani), Male, Taxi driver).*

### Acceptability of other emission reduction and travel and transport management activities

In addition to the CAZ the groups explored the acceptability and feasibility of 12 additional emission reduction, or travel and transport management activities which could be considered to complement the CAZ and further improve air quality. A summary of opinion towards these activities can be found in Table 2. Activities such as park and ride, highways improvements and upgrading the councils’ fleet to less polluting vehicles were viewed positively by all participants. Activities such as increasing parking charges in public car parks, improving cycling infrastructure and planning and development initiatives had a mixed response, participants were less supportive of initiatives where they would be personally inconvenienced or affected.

**Table 2:** Summary of participants reactions to acceptability and feasibility of additional emission reduction, and transport and travel management activities

**Emission reduction initiatives**	
**Green buses scheme**	Although participants felt that this would be a good initiative, there was some scepticism about whether the costs incurred by bus companies would then translate into higher fare prices for consumers. In the absence of that, it was argued that this would be a good initiative. *“It’s a really good idea but If it costs them x amount of money to get new buses all they are going to do is put the fares up to cover that cost then who loses out? It’s us again, the public” (FG10- Male).*
**Installation of electric charging infrastructure in new developments**	Participants struggled to grasp this idea. They felt that it was too innovative and not feasible and would not influence their purchasing habits in a free market. *“I’m not gonna buy a house just because it’s got a charging point outside of it. I’d be worried about it blowing up. It would probably put me off if anything” (FG7- British Asian (Pakistani), Male, Unknown profession).*
**Council fleet**	Most people felt that the council should introduce greener vehicles in their fleet however again, some felt that it could ultimately lead to an increase to their council tax and burden and individual residents and consumers further. *“It’s a good starting point but the Council’s skint here. They will somehow find a way to put that into your Council Tax but I suppose it helps to lead by example” (FG3- British Asian (Pakistani), Female, Office worker).*
**Travel and transport management**	
**Park and ride**	This was seen as a workable and positive initiative by most participants. *“I think that’s a really good idea”. Just make it super cheap and people will do it … Like really it has to be like really cheap” (FG1- British Asian (Indian), Female, Retired).*
**Road widening/ highways improvements**	There were mixed feelings about this. Some people felt that it would encourage more driving (not less) and restrict pavement space for pedestrians. *“If you try and widen the road as you are suggesting isn’t that going to be an invitation for more cars to come into Bradford? It wouldn’t be good if they narrowed the pavement for the people walking” (FG2- British Asian (Pakistani), Female, Housewife).* It was a popular initiative amongst drivers who felt that it was a good idea and it would help reduce idling and congestion. *“There are some roads that are so narrow that if traffic builds up there you can be stuck there for ages. Just think of the pollution for the people who live on that street” (FG1- British Asian (Other), Male, Bus driver).*
**Intelligent transport systems**	Some people felt that this initiative would be ineffective due to poor driving habits by some. They argued road users would intentionally ignore traffic lights or that it would encourage them to speed up if they knew the lights were about to change. *“Because I think when people, especially in Bradford, young people driving through red light, it will happen more like this” (FG3- British Asian (Pakistani), Female, Retired).*
**City centre parking demand charges**	Most people were not in favour of this option as they felt that it would negatively impact on the businesses in the city centre. People would be encouraged to go to other shopping centres where there was cheaper or free parking and there was scepticism about whether this measure would make any significant contribution to improving air quality. *“You could increase the cost, it will not reduce pollution. People will get dropped off or they’ll park in a free car park. You can park in Tesco for a couple of hours. I think it would affect the economy as it is anyway, so that’s a massive no-no. That’s not going to work. I don’t think so anyway” (FG3- British Asian (Pakistani), Female, Teacher).*
**Freight consolidation centres**	Most people felt that this wasn’t a suitable initiative as it would cause numerous disruptions with little benefit. Participants were also unsure about how this initiative would be operational with concerns about increased traffic. *“if you’ve got frozen goods on that particular lorry, you’re going to put it into the transport in ten different, are you going to tell me they’re going to employ two or three people more to put it into the van and then unload it out, it does not work” (FG7- British Asian (Unknown), Male, Youth worker).*
**HGVs- traffic diversions and delivery times**	Most people agreed that having time restrictions on when HGVs can use the highways would be a useful measure. Many felt that it would also help reduce congestion on the roads. *“And within certain hours people will probably go out less anyway because they know these guys are out on the roads, so give them that time and it would help traffic flow better … I don’t think they should be out in busy times” (FG5- (British Asian (Pakistnai), Female, Housewife).*
**Travel planning**	Most people argued that they would car share if it was feasible for them and dependant on who they shared with and where they intended to go. *“There’s a car share thing, a scheme that people do. I would do with work colleagues” (FG3- British Asian (Pakistani), Female, Office worker).*
**Cycling and cycling infrastructure**	There were mixed feelings on cycle lanes. Some people felt that cycle lanes have been were being misused and therefore not correctly utilised enough. *“You’ve got the cars that park on the cycle lanes, and delivery men” (FG1- White British, Male, Retired).* They also felt that poor driving habits by some road users made them feel unsafe to cycle. *“And dangerous driving from others … Yeah. So there’s a lot of issues with the cycling” ((FG3- British Asian (Pakistani), Female, Community support worker).* Some people felt that the landscape in Bradford meant that cycling as a mode of transport would be difficult for a lot of people and it was more suited as a leisure activity. *“Cycles are okay in York and Nottingham and London, where it’s all one level flat levell, Halifax, Bradford, here, uphill and down it’s a bit of a struggle” (FG1- British Asian (Other), Male, Bus driver).*

### Acceptability and readiness for electric vehicles

Most participants expressed concern about the affordability of electric vehicles. *“They cost between 20 and 30,000 quid don’t they? “I haven’t got the funding for it, you’ve got to understand we don’t have the funding for it” (FG4- British Asian (Pakistani), Female, Housewife).* However, some did recognise that although the initial cost is higher, this might not be the case long-term; *“they’re like a diesel car, so it’s a case of the initial outlay is more. In the long-term it will get cheaper” (FG3- British Asian (Pakistani), Female, Community support worker).*

Further, it seemed that some people would be open to owning an electric vehicle should they become affordable or financial support become available.

*“I mean the government have got to look a bit serious as well though, I mean if they can fund out money then people will think more of it, if the government is helping us, you know, we will have, like you said here, electric cars, I will drive an electric car if I get some funding towards it” (FG7- British Asian (Pakistani), Male, Unknown profession).*

There was some mistrust amongst participants about information suggesting that electric vehicles are cleaner and greener. Participants highlighted that in the recent past diesel cars were being promoted as being more economical than petrol and thus cleaner. This led to many people investing in diesel vehicles. This myth was busted as a result of the diesel emission scandal (also referred to as ‘dieselgate’) scandal which erupted in September 2015, [39] and many people are therefore now reluctant to change again.

*“You must have heard it, the Council said, what did they say were the best cars, the diesel cars, yeah, they said buy the diesel cars, this and that, I went out and bought myself a diesel car, thinking because it’s better than the petrol, now they come up and say diesel cars we’re going to scrap it, excuse me, but I just bought a new car, and it’s on diesel because you told me to get it and now you’re saying get rid of your diesel cars, get rid of your petrol cars, move to electrical, I’m really actually annoyed about that (FG4- British Asian (Pakistani), Female, Housewife).*

This highlights a further reason why some felt cautious about making an investment in electric vehicles as some felt that information and guidance changes more often than they can afford for it too.

Some participants suggested that hybrid vehicles would be a better option than fully electric vehicles. This was particularly favoured by taxi drivers as they would not be restricted when there was a need for recharging. *“The hybrid is better but you’re still using petrol or whatever it runs on but its better, it’s more sensible than having an electric car” (FG7- British Asian (Pakistani), Male, Taxi driver).*

Most people felt that if there was an incentive for people to trade in their vehicles for greener vehicles then they would be more receptive to the idea. *“To say like if you trade in your diesel car for like an electric car then, you know, we’ll give you 10 percent off” (FG3- British Asian (Pakistani), Female, Teacher).*

Although it was recognised that there can be a financial as well as environmental benefit in the long term, for many people in these communities, the initial financial barrier was a key component of their decisions and driver towards changing behaviours and in this instance, changing their vehicles.

### Public transport and active travel

All participants felt that public transport in the area was too expensive. It was felt that fare prices need to be reduced and buses need to be more accessible in order to get people to make a shift towards using public transport for their travel purposes. People felt that buses, in particular, were too expensive. *“[Public] transport is so expensive. It’s cheaper for people to actually go somewhere in a car than it is to go on the bus. If they want people to use public transport, they have to, you know, look at the cost element to it” (FG6- British Asian (Pakistani), Female, Community support worker).* Participants highlighted that high public transport fares will mean “*less people use them”* so the initiative may *“backfire because its unaffordable. It’s almost forcing people not to use the buses” (FG7- British Asian (Pakistani), Male, Unknown profession).*

Additionally, some participants felt that buses were infrequent and did not offer direct routes to many parts of the city. This can ultimately make a short journey problematic in a bus if all journeys require a route change through the city centre:

*“I only use public transport occasionally, but they’ve chopped so many different routes off their system that some places I want to go I either have to for example, if I wanted to go say from this set of traffic lights up to the other set of traffic lights, I have to go into town, get another bus to go up to get to that, because this one they’ve taken off, so this one that used to go what they used to call the city circle went from here all the way around Bradford, past St Luke’s Hospital, so you could get off go to your hospital appointments, get back on again but they’ve taken in off” (FG1- White British, Female, Retired).*

Some people highlighted issues with accessibility of public transport particularly for vulnerable groups:

*My mum who’s got limited walking abilities at the moment, and the buses, some where they have got lowering floors but they literally do not lower down as properly as they should. So you’re talking people who have got disabilities, wheelchairs, prams, like again if you want us to use the public buses, transport and things, make it adaptable for people. I’ve still seen buses that they can do a lot more than what they are doing now” (FG5- British Asian (Other), Female, Retail worker).*

It is clear that there is an appetite for more use of public services and with better connections, improved services and cheaper fares that more people would consider travelling by bus, rail or other modes of public transport.

Some participants felt that there should be more school buses to local schools within their neighbourhoods. This will reduce the amount of traffic on the roads as parents dropping and collecting their children currently contributes to a significant amount of traffic during peak times. *“The solution like, you know, there’s so many schools in Bradford, they should run some buses for school. This is a better idea because it’s like 500-700 kids and probably 250 people coming with the car and the rest of us are walking. Imagine how many cars are there” (FG2 British Asian (Pakistani), Female, Community support worker).*

### Behavioural changes

There was a suggestion that developing a programme for behavioural change techniques along with the CAZ to support communities was needed for its long-term success.

Many participants felt that changing narratives with children to develop new behaviours would lead them to promoting active travel amongst their families for a greener future. *“Promote it in a good way for the children, get them to start riding and as they get older they might not want to get a car and they might want to just get a bike instead” (FG5- Female).*

*“I think the best way to do is teach it in schools because once the kids are on it they’re going to be badgering mum and dad at home, they’re going to be like ‘oh we can’t drive, we have to go walking to the Co-op because, you know, it’s air pollution and fifty thousand people a year die‘ and, you know, it needs to be a big marketing campaign where it’s in the schools, it’s on the buses, everywhere you go. And the more people are aware of it that will encourage people to change their habits” (FG3- British Asian (Pakistani), Female, Teacher).*

One participant gave the following example about how prone they were to relapse. *“I think it’s a big part is the weather, that puts you off mostly, I mean I’ve got a bike and I think, oh I’d like, but then as soon as it’s windy, it’s rainy, it’s cold, and you think, do you know what, just get in the car anyway” (FG1- British Asian (Other), Male, Bus driver).*

There were some participants who were unsure about the impact of individual action. They felt that efforts need to be on a citywide or nationwide level in order to achieve impact. They felt that for them to make changes individually would not have a significant impact if then other members of their community or the wider community did not also adopt changes. This would render their efforts insignificant.

It was nevertheless noted that behaviour change is deliverable when penalties are included in plans. This was considered to be the case with the introduction of a 5p charge for plastic carrier bags which immediately saw a significant reduction in use [40]. Some participants suggested that if a charging CAZ was introduced, then people would come to accept it and make the necessary changes. *“You know, people do complain and there will be a lot of like uproar about it but I think they’ll just expect it after a bit. It’s like the bags, with the shopping bags” (FG4- British Asian (Pakistani), Female, Housewife).*

Participants suggested that more resources need to be invested in challenging some taboos within their communities. For example, they argued that, few people within the South Asian community choose to cycle. There were a number of suggested reasons for this, from safety concerns to cultural inappropriateness amongst females. Nevertheless, participants felt that these stigmas could be overcome. *“Let’s make cycling kind of normal. Take big groups out and make it normal, where they are riding and then it becomes more and more popular and people want to do it” (FG3- British Asian (Pakistani), Female, Teacher).* Further work is required in areas where there are particular cultural sensitivities.

## Discussion

With ever expanding media coverage, air quality is a prominent issue both in the UK and globally. Amongst our low-income, multi-ethnic sample we found there was widespread knowledge and awareness about the importance of air quality for health and wellbeing. However, we found that air quality was often prioritised less than other more ‘visible’ environmental concerns. We found there was little consensus about which type of CAZ class (A-D) would be most acceptable to implement to tackle poor air quality. However, we found a clear a mistrust of these proposals which were felt would disadvantage already deprived communities the most.

To some extent divergence in opinion was influenced by people’s circumstances. For example, non-drivers favoured more stringent CAZ classes (for example those targeting private vehicles), those with cars favoured less stringent CAZ classes (where private vehicles were exempt). For people whom would be most financially affected by a CAZ (e.g. taxi driver) the implication of the CAZ for their livelihood trumped any concerns about air quality.

There was clear concern amongst all study participants about the potential for significant financial burden associated with a range of initiatives which would be felt more strongly by already deprived communities; for example, with increased costs for local businesses and transport operators being passed to consumers and commuters. Taxi and hackney cab drivers were identified as a particularly vulnerable, low-income group who would be disproportionately affected by all potential CAZ classes, and who may lack the ability to upgrade to standards. If charging CAZ are to be implemented, then consideration of how to mitigate adverse economic impacts for this group will be crucial to ensuring inequalities are not increased.

Amongst our sample, there appeared to be high levels of mistrust about the motivation behind initiatives to improve air quality, with some suggestion that local government’s main benefit would be a financial gain from penalties. For many, more visible environmental concerns within their community (for example, fly-tipping) were seen as higher priority concerns in comparison to ‘invisible’ pollution. These concerns meant that many participants viewed the CAZ proposals with a sense of mistrust, viewing them as ‘money-making’ tactics for the council rather than initiatives which would bring benefit to their communities. This emphasises the importance of retaining a holistic view of environmental poverty when developing new policy. Research suggests that poorer communities are disproportionately exposed to a range of environmental hazards [41]. Thus, trying to encourage people to truly accept and adopt changes for an invisible issue such as poor air quality up against more visible challenges may be difficult if the latter are also not addressed.

Participants were keen to convey the message that any proposals should not solely affect individuals from low socio-economic backgrounds and that businesses in the area should share the burden. Participants in our study were able to suggest a wide range of suggestions about how to improve air quality. While some suggestions would not be feasible or practical to implement from a policy perspective, it is important that policy makers understand community preferences so that they can devise appropriate communication and engagement activities to help work together to achieve acceptable and practicable solutions.

It is clear from this research that poor air quality and very high levels of pollution in parts of the UK that are disproportionately disadvantaged in socio-economic terms is a matter of concern for the community. However, faced with multiple disadvantages already, it is important that any initiative to help improve air quality does not unduly have a negative effect on deprived communities. If communities are seldom heard then they may not be able to effectively engage with decision makers to influence policy to minimise unintended consequences. Equity focused health impact assessment (EFHIA) [42] is one tool that which could provide useful in identifying potential inequities and therefore mitigate against this in policy decisions. EFHIA uses health impact assessment methodology in a structured way to explore the potential differential impacts of a proposal on the health of specific groups within a population and to assess if these differential impacts are inequitable”, [43] in a way that can be absent or inconsistent in traditional health impact assessment methods [44]. It involves the following steps: 1) screening: determining the suitability of the topic, identifying populations likely to be affected and key stakeholders; 2) scoping: clarifying the dimensions of equity that are of interest (e.g. access, resources, outcomes), agreeing definitions and outcome measures and agreeing the process for the EFHIA; 3) impact identification: searching literature for evidence and conducting stakeholder consultations to identify potential or actual impacts, and critically appraising this evidence; 4) assessment of impacts: weighting and synthesising evidence, identifying nature and likelihood of equity impacts; 5) recommendations: recommending changes based on identified likely equity impacts and links to health; and 6) monitoring and evaluation: developing and implement strategies for monitoring update and impact of EFHIA and outcomes of the implemented policy [42].

Of course, EFHIA will only be effective if those conducting assessments can effectively identify, engage and listen to disadvantaged groups. In the context of CAZ, we have identified taxi and private hire vehicle drivers as particularly disadvantaged, in addition to other groups living in deprived communities; these will be important groups to include when developing clean air proposals.

Participants in our study also discussed initiatives that were reliant on individual behaviour; for example, upgrading to electric cars and increasing use of public transport. Not surprisingly, perceived barriers to more widespread uptake of electric vehicles were mainly financial, with costs being too high to make this a viable option for communities living within deprived areas. Financial reasons were also cited as a barrier to using public transport or engaging in active travel, along with structural factors such as the availability of services, and safety of the environment for cycling.

### Strengths and limitations

Our study had a number of strengths. We recruited a large, diverse, multi-ethnic sample who were living within deprived parts of Bradford district. We are able to include a range of younger and older participants and non-English speakers using familiar and accessible venues. Many of these communities are considered ‘seldom-heard’ or ‘under-researched’ and by including their voices in this research we were able to understand potential unintended consequences of implementing air quality policy among vulnerable groups. However, our sample may have suffered some element of self-selection bias in that individuals more interested in and aware of air quality issues may have agreed to attend. We were not able to record systematically characteristics of people who were approached to take part but declined. Our sample was predominantly of Pakistani origin, as such our findings may be less relevant to more affluent or less ethnically diverse settings. Whilst our researchers were able to conduct field work in English and Urdu languages, we relied on use of an interpreter for our Eastern European Roma focus group. This may have impacted on dynamics and understanding.

Our participants reported a generally good awareness about poor air quality, but there was a perception amongst some that others in their community may not perceive it as an issue for concern. However, this may reflect a systemic tendency of participants to underestimate feelings within communities on issues that they may be less familiar with [45].

### Recommendations for policy and practice

Based on our findings, we propose the following recommendations for areas planning to implement city wide interventions to improve air quality:

1. That an equity focus health impact assessment is carried out to ascertain any potential unintended or adverse consequences that may increase health inequalities.
2. That multiple methods are used to engage with seldom heard populations and disadvantaged groups that may be adversely affected by proposals, including in-depth interviews, focus-groups as well was surveys and consultation events.
3. In relation to implementing charging clean air zones:
  - Development of a mitigation strategy to ensure that low-income taxi drivers are not economically disadvantaged by CAZ proposals; and that any strategy is developed in partnership with representatives from this community.
  - Development of a mitigation strategy to ensure the costs incurred by public transport operators are not passed on to low-income consumers.
  - In relation to CAZ D: Development of a mitigation strategy to ensure that low income families who own older, non-compliant vehicles are not disproportionately economically disadvantaged by CAZ proposals.
  - Mitigation strategies for above could include provision of grants to allow recovery of costs associated with upgrading vehicles to compliant standards, or to allow local exemptions.
4. That the potential impact of other emission reduction or transport management activities on the ease of walking and cycling be explicitly considered to ensure that these activities do not have a detrimental impact on pedestrians and cyclists.
5. Development of an accessible community engagement strategy aimed at low income, seldom heard communities is needed to raise awareness of air quality issues and need for initiatives such as CAZs to be implemented to improve air quality and health. This should acknowledge that air quality may not be the number one priority in some deprived areas where there are other environmental concerns (such as fly-tipping and unclean streets).

## Conclusion

City wide interventions such as Clean Air Zones have the potential to quickly improve air quality in polluted areas; however, may have unintended impacts on low-income, deprived communities. The key messages emerging from this research were twofold. First, that the financial burden of funding these initiatives or a later consequence of the proposals should not fall on already deprived communities. Second, that vulnerable and seldom-heard communities need to be widely consulted and engaged with when developing proposals with a view to identify potential impacts and on health equity and strategies to mitigate against these. This engagement will create a sense of ownership of a programme with communities which will ultimately lead to a wider acceptance of it. Policy makers wishing to implement specific initiatives to improve air quality need to understand the wider context of environmental injustices that deprived communities face and develop coherent strategies to address wider concerns if initiatives are to be implemented successfully.

## Supplementary Information

**Additional file 1.**

## Data Availability

The transcripts analysed during the current study are not publicly available due to confidentiality but are available from the corresponding author on reasonable request.

## Abbreviations

CAZ: Clean Air Zone
COPD: Chronic Obstructive Pulmonary Disease
DEFRA: Department for Environment, Food and Rural Affairs
FG: Focus Group
LEZ: Low-Emission Zone
NICE: National Institute for Clinical Excellence
NO2: Nitrogen Dioxide
PM: Particulate Matter
WHO: World Health Organisation
