# Supplementary material for: Taking a deep breath: a qualitative study exploring acceptability and perceived unintended consequences of charging clean air zones and air quality improvement initiatives amongst low-income, multi-ethnic communities in Bradford, UK
Source: BMC Public Health. 2021 Jul 3;21:1305. doi: 10.1186/s12889-021-11337-z (PMC8255006; doi:10.1186/s12889-021-11337-z)
Supplement: Supplementary file 1 — Additional file 1. [file 12889_2021_11337_MOESM1_ESM.doc]

Supplementary File 1

**Table 1 Focus group demographics and ward characteristics**

| **Focus Group** | **FG1** | **FG2** | **FG3** | **FG4** | **FG5** | **FG6** | **FG7** | **FG8** | **FG9** | **FG10** | Total |
| --- | --- | --- | --- | --- | --- | --- | --- | --- | --- | --- | --- |
| **Location** | Laister-dyke | Girlington | Shipley | Little Horton | Under-cliffe | Keighley | East Bowling | Barker-end | Thornbury | Thornbury |  |
| **Ward** | **Bradford Moor** | **City** | **Shipley** | **Little Horton** | **Bolton & Under-cliffe** | **Keighley Central** | **Bowling and Barkerend** | **Bowling and Barkerend** | **Bradford Moor** | **Bradford Moor** |  |
| **Ward deprivation rank^a^** | 4 | 7 | 21 | 2 | 15 | 6 | 3 | As FG7 | As FG1 | As FG1 |  |
| **Top two ethnic groups in ward** | 64% Pakistani  17% White | 43% Pakistani  25% White | 7% Pakistani  84% White | 49% Pakistani  29% White | 14% Pakistani  67% White | 43% Pakistani  43% White | 33% Pakistani  43% White | As FG7 | As FG1 | As FG1 |  |
| **% in Ward aged**  Under 16  16-64 | 32%  61% | 22%  72% | 19%  64% | 33%  60% | 25%  62% | 27%  60% | 30%  62% | As FG7 | As FG1 | As FG1 |  |
| ***Breakdown of participants in focus group*** | | | | | | | | | | | |
| **Number of participants** | 6 | 18 | 5 | 12 | 11 | 6 | 7 | 9 | 10 | 3 | 87 |
| **Age (years)** |  |  |  |  |  |  |  |  |  |  |  |
| 20-30 | 0 | 0 | 0 | 6 | 0 | 1 | 3 | 2 | 1 | 1 | 14 |
| 30-40 | 0 | 0 | 4 | 6 | 3 |  | 3 | 5 | 6 | 0 | 27 |
| 40-50 | 2 | 3 | 0 | 0 | 6 | 3 | 1 | 2 | 2 | 1 | 20 |
| 50-60 | 1 | 5 | 0 | 0 | 1 | 2 | 0 | 0 | 1 | 1 | 11 |
| 60+ | 3 | 10 | 1 | 0 | 1 |  | 0 | 0 |  | 0 | 15 |
| **Gender** |  |  |  |  |  |  |  |  |  |  |  |
| Male | 3 | 16 | 0 | 0 | 0 | 0 | 7 | 9 | 0 | 0 | 35 |
| Female | 3 | 2 | 5 | 12 | 11 | 6 | 0 | 0 | 10 | 3 | 52 |
| **Ethnicity** |  |  |  |  |  |  |  |  |  |  |  |
| Asian Bangladeshi | 0 | 0 | 0 | 0 | 0 | 2 | 0 | 0 | 0 | 0 | 2 |
| Asian Indian | 2 | 0 | 0 | 1 | 0 | 0 | 0 | 0 | 1 | 0 | 4 |
| Asian Pakistani | 1 | 18 | 5 | 11 | 10 | 4 | 6 | 9 | 7 | 0 | 71 |
| White English | 2 | 0 | 0 | 0 | 0 | 0 | 0 | 0 | 2 | 0 | 4 |
| White European | 0 | 0 | 0 | 0 | 0 | 0 | 0 | 0 | 0 | 3 | 3 |
| Other (Arab/Mixed) | 1 | 0 | 0 | 0 | 1 | 0 | 1 | 0 | 0 | 0 | 3 |

Note: Ward details taken from the 2011 Census: <https://ubd.bradford.gov.uk/district-profiles/ward-profiles-2020/>, accessed 8^th^ April 2021

^a^ number reported is the rank of most deprived ward in the district, out of 30 wards

Supplementary File 2: **Topic Guide used in Focus Groups**

**General Views**

1. What do you think about the air quality in your area? Can it be better?
2. How important do you think the issue of clean air is to people?
3. What are your feelings about it?
4. What do you think causes pollution outside the house?
   1. Prompt: types of vehicles inc HGVs
5. What do you think causes pollution inside the house?
   1. Prompt: old boilers/ wood burning stove

**Health concerns**

1. What health conditions do you think are of most concern at the moment?
2. Who is most vulnerable to air pollution?
   1. Prompt: elderly/ children
3. How serious do you think the problem is?

**Proposals**

1. What do you think can be done to improve air quality that would be acceptable to people and is doable?

There are 4 CAZ proposals *(show visuals and explain [*government recommend Clean Air Zones to reduce pollution – this could involve charging difference types of vehicles to enter]

1. Which CAZ option would you prefer? Why?
2. Can you foresee any barriers/ potential problems to implementing the CAZ?
3. Are there any negatives related to implementation?
4. The CAZ’s affect different groups such as bus companies, HGVs/ LGVs, private hire. How would these groups feel about being charged?
5. What else can be included in CAZ?
   1. Emission reduction activities*(show visuals)*
   2. Transport and traffic management activities *(show visuals)*
6. How can change be facilitated? What would people be happy to adopt? What will they be resistant to?
7. How will we know if CAZ’s are working?
8. Do you think there are other benefits to CAZ measures other than better air quality?
9. What do we need to do to ensure everyone’s opinion is heard?

*Now I want to talk about some different activities*

**1) Anti-Idling**

1. Do you know what anti idling is? *(Explain and say that lots of schools want to tackle this)*
   1. How much of a problem do you think it is?
2. Do you think people need more education around this?
   1. What would help enable this?
   2. What would be barriers towards this?
3. Do you think people will be willing to change their driving habits?
4. What do you think about low emission cars? Will people change their cars in favour of low emission vehicles?
   1. Prompt: Cost/ desirability

**2) Electric Cars**

1. What do you think about electric cars?
2. What do you think will encourage people towards electric cars?
3. What do you think are the barriers?
   1. Prompt: availability of charging points/ costs/ desirability

**3) Public Transport**

1. What do you think about current public transport links in this area?
   1. Prompt: Trains/ buses/ park and ride
2. How accessible is public transport?
3. What would encourage more people to use public transport?
4. What are the challenges to using public transport?

**4) Active Travel**

1. What are your thoughts on cycling/ cycle lanes?
2. Do you think more people will be willing to take up cycling as a mode of transport?
3. What will help change people’s behaviours such a walking for short journeys?
   1. Prompt: School run
4. What are barriers to this?
5. What other types of active travel measures can you think of?

**Concluding thoughts**

1. Is air quality high enough on people’s agenda to want to make a personal change?
2. Any other comments/ suggestions?
